# Variation of Relief Topography and Hardness of Surface Layers of Materials Due to Impact-Oscillatory Loading

**DOI:** 10.3390/ma12172720

**Published:** 2019-08-25

**Authors:** Mykola Chausov, Pavlo Maruschak, Andrii Pylypenko, Janette Brezinová, Roman Bishchak, Iurii Burda

**Affiliations:** 1 Department of Mechanics, National University of Life and Environmental Sciences of Ukraine, Heroiv Oborony str. 15, 03041 Kyiv, Ukraine; 2 Department of Industrial Automation, Ternopil National Ivan Puluj Technical University, Rus’ka str. 56, 46001 Ternopil, Ukraine; 3 Department of Engineering Technologies and Materials, Faculty of Mechanical Engineering, Technical University of Košice, Mäsiarska 74, 04001 Košice, Slovakia; 4 Department of Welding, Ivano-Frankivsk National Technical University of Oil and Gas, Ivano-Frankivsk, Karpatska str. 15, 76019 Ivano-Frankivsk, Ukraine; 5 Empa, Swiss Federal Laboratories for Materials Science and Technology, Laboratory for Mechanical Systems Engineering, Überlandstrasse 129, CH-8600 Dübendorf, Switzerland

**Keywords:** mechanical characterization, aluminum alloys, plasticity, titanium alloys, dynamic non-equilibrium processes

## Abstract

It was shown previously that cyclic loading can be used to extend the fatigue life of sheet plastic materials subjected to the preliminary impact-oscillatory loading. This type of loading causes dynamic non-equilibrium processes (DNP) in materials, which lead to the formation of dissipative structures in materials and on their surface. The density of these dissipative structures is less than that of the base metal. In this paper, the results of investigations into the relief and hardness of surface layers modified by impact-oscillatory loading are analyzed on the example of five structural materials. The signs of a regular, orderly system of microextrusions formed on flat surfaces of all materials due to DNP are considered along with the alignment of roughness parameters R_z_ and R_a_ of relief profiles. The effect of impact-oscillatory loading is one of the main causes that lead to the extension of the fatigue life of materials.

## 1. Introduction

Experimental investigations into the mechanical behavior of plastic materials under dynamic non-equilibrium processes (DNP) require further study and generalization of material deformation patterns [1,2,3,4,5]. At the same time, structural transformations that occur in a material under DNP are considered using the methods of physical mesomechanics. These methods presume the presence of multiscale carriers of plastic deformation and consider the thermodynamics of non-equilibrium processes [5,6,7]. As a result, new possibilities are provided for upgrading the machining technology of materials used in the manufacture of load-bearing structures of transport systems. The multiscale material analysis makes it possible to link deformation conditions and changes in hardness and roughness of the deformed material. In addition, the revealed deformation patterns can be systematized, and the durability of load-bearing structures can be predicted [8,9,10].

Sheet titanium and aluminum alloys, as well as stainless steels, are widely used for the manufacture of modern aviation and rocket structures. During operation, they are subjected to complex types of loading, in particular, cyclic ones [11,12,13,14]. Therefore, the task of extending the fatigue life of such materials is very relevant. It is known that energy fields, such as laser, electromagnetic, ultrasonic and other fields, change the initial physical and mechanical properties of materials, in particular, those of their surface layers [15,16,17,18,19,20]. It was found that dynamic non-balanced processes (DNP) arise in materials under pulse influences. This causes significant changes in the initial mechanical properties of structural materials. While analyzing the results of previous studies, it should be noted that they were conducted using complex and expensive equipment without attaining a significant mechanical effect. Therefore, their physical justification cannot be adequate [15,16,17,18,19,20].

It should be emphasized that practically no data has been reported in literature on the realization of dynamic non-balanced processes in materials due to the influence of force fields, which are created by additional force impulse loading. Changes in the mechanical properties of materials and their surface layers due to additional force impulse loading applied under DNP directly affect the workability and residual life of many critical structures that operate under variable loads.

A new effective method for modifying the initial mechanical properties of materials by means of impulse introduction of power energy under impact-oscillatory loading should be developed and tested. This will make it technologically easier and cheaper to improve the physical and mechanical properties of materials and their surface layers [21,22,23]. Plastic deformation, impact toughness and crack resistance of materials can be enhanced significantly under loading applied after the DNP [21,22,23]. In addition, with further cyclic loading it is also possible to extend the fatigue life of materials [24,25,26].

Sophisticated physical methods were used to study real dissipative structures formed in materials after DNP. The density of these dissipative structures is less than that of the base material. Thus, aluminum alloys are characterized by newly formed dissipative structures in the form of thin-layer structures interconnected at different structural levels [27,28]. Two-phase titanium alloys are characterized by the mechanisms of structure fragmentation [29,30,31,32].

Particularly noteworthy is that this process can be controlled, and its parameters can be regulated by changing the intensity of the impulse introduction of energy into the material at room temperature [22].

Previously, attempts were made to physically substantiate the revealed mechanical effects of impact-oscillatory loading on extending the fatigue life of materials. In particular, to study the kinetics of changes in the surface layers during the impulse introduction of energy into the aluminum alloy D16ChATW, a special monocrystalline sensor was firmly fixed with a Pasco Fix adhesive, (PASCO Industrial Adhesives, Freilassing, Germany) on the surface of specimens. After that, shooting was performed. The rate of shooting the specimen surface during impact-oscillatory loading was 600 fps. The form of relief was considered as one of the parameters that allow estimating the effect of impact-oscillatory loading on the surface condition of the material [23]. In the process of DNP caused by impact-oscillatory loading, microextrusions and less dense dissipative structures were detected on the surface of a single-crystal sensor. They indicate changes in the structure and mechanical properties of the alloy, and, most importantly, in its surface layer. The resulting hybrid surface structure is characterized by the alternation of soft and solid microzones. As a result, microcracks are arrested on the specimen surface during cyclic loading, and the fatigue life of aluminum alloys is extended. The data obtained are predominantly qualitative, however, they indicate the prospects of such studies for assessing changes in the topography of the surface layers of materials under DNP. Moreover, the revealed mechanical effects can be explained using more contemporary methods. Studies have not been conducted to evaluate the hardness of surface layers of materials after DNP caused by impact-oscillatory loading. Such studies would confirm the dominant influence of dissipative structures formed in materials under DNP on changes in the hardness of surface layers.

It should be noted that similar research by other authors was also focused on changes in the surface roughness of aluminum and titanium alloys in the process of large and superplastic deformations. However, it was shown that in the case of static loading (SL), the surface roughness of materials increases with an increase in the deformation level and depends on the structure of the grain size [24,25,26].

The purpose of this research was to evaluate the relief topography of surface layers after dynamic deformations caused by DNP in materials of different classes, and to evaluate the hardness of surface layers of materials after DNP, as compared to the effect of the static load at the same level of deformation.

## 2. Methods of Mechanical and Physical Research

**Figure 1:**
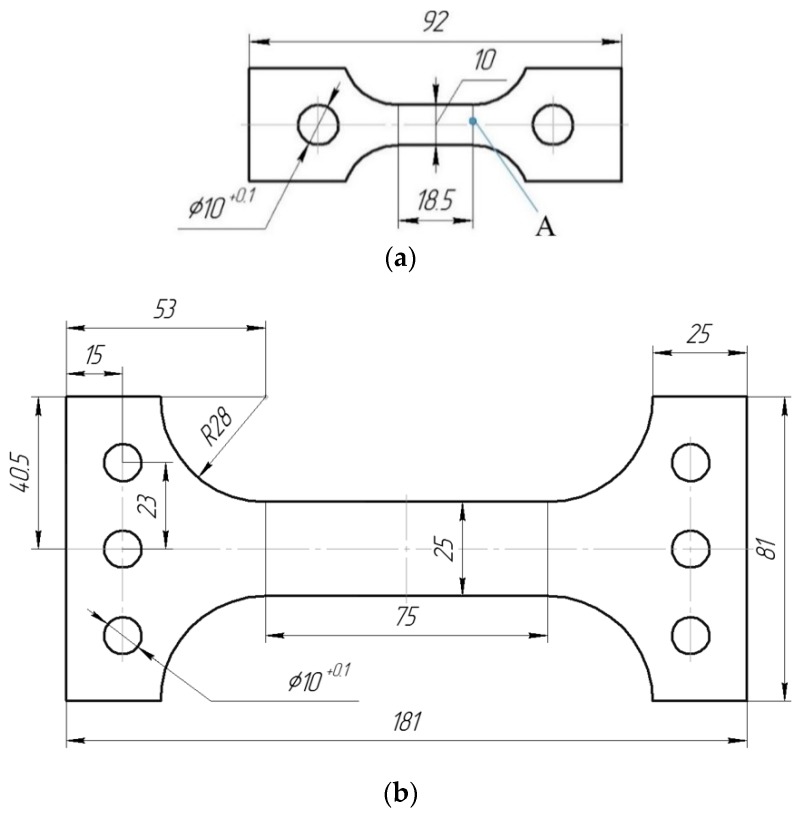
Test specimens: (**a**) Small specimen; (**b**) large specimen.

Tests were performed on sheets of industrial aluminum alloys D16ChATW and 2024-T351, two-phase (α + β phase) titanium alloys VT23 and VT23M, and stainless steel 12Kh17 with a thickness of 3 mm. Only one sheet of each material was used for making specimens. To evaluate the topography of surface layers, specimens (Figure 1a) from alloys D16ChATW, 2024-T351, VT23, VT23M were tested. The hardness of the surface layers was evaluated on specimens from steel 12Kh17 (Figure 1b).

One side of all specimens (Figure 1a) was polished before testing using the same technology. The strain measurement base was 16 mm. The impact-oscillatory loading was applied on a modified hydraulic installation for static tests ZD-100Pu (WPM, Leipzig, Germany). Its main methodological aspects are described in detail in [27,28,29]. The basic idea of the proposed technique was to apply impact-oscillatory loading during high-speed tensioning of materials, with a high frequency (several kilohertz) oscillatory process overlaid, which corresponds to the own frequency of the test machine. The power energy of different intensities was introduced into the material after the fracture of two brittle satellite specimens with circular concentrators. The satellite specimens are made of hardened steels of different cross sections for a given level of strain or a given load applied to the specimen. A high-speed camera Phantom v 711 (Vision Research Inc., NJ, USA) with a recording speed from 15,000 fps to 680,000 fps was used to evaluate the strain rates of aluminum alloys and stainless steel at the stage of the formation and development of dissipative structures in materials [29]. The amplitudes of the vibrational load in the process of DNP are also given.

As is known, there are several possible practical variants to control the impulse introduction of power energy into materials under impact-oscillatory loading [22]. In this research, the impulse introduction of energy into materials was done as follows. Three specimens from each of the four materials under study were statically loaded to the same level of force loading (2.0 kN). Then, they were subjected to the same impulse force influence of 42.0 ± 2 kN and were completely unloaded immediately.

A sudden increase in the strain under the impulse introduction of energy—*ε_imp_*—was chosen as a parameter that characterizes the intensity of the impulse introduction of energy into the materials. The choice of *ε_imp_* as a parameter that characterizes the intensity of the impulse introduction of energy into the alloys greatly simplifies the test procedure under impact-oscillatory loading. In this case, the modes of impact-oscillatory loading can be created using the hydraulic test machines of varying rigidity. This eliminates the need for complex calculations of a particular force influence applied to the specimen depending on the total pulse applied to the mechanical system. In addition, such procedure is intended for real technological processes.

Changes in the topography of the polished flat surfaces of specimens in the initial state and after DNP were evaluated using the 3D Optical Surface Metrology System “Leica DCM8” (Leica Mikrosysteme Vertrieb GmbH, Wetzlar, Germany) for the reliability of research, the relief parameters were compared in the central regions of specimens (zone A in Figure 1a).

To evaluate the hardness of the surface layers of materials in the initial state and after DNP, specimens (Figure 1b) from steel 12Kh17 were used. Steel 12Kh17 was chosen for research based on the following assumptions. Firstly, all additional impulse loads were realized on the ascending branch of the stress-strain diagram without any signs of a “neck”. Secondly, the largest strain range of the material under DNP was covered. Given this, steel 12Kh17 has the longest section on the stress-strain diagram that describes the stage of strengthening compared with other materials under study. Thirdly, this steel was used previously to study changes in the mechanical properties of materials caused by DNP. This made it possible to compare the results obtained [32,33]. The strain measurement base was 75 mm. The hardness of the surface layers of steel was measured using a portable hardness meter Computest SC (Qualitest International Inc., Lauderdale, FL, USA) under the load of 5 kg.

Mechanical properties of materials are given in Table 1. The chemical composition of the materials is presented in Table 2.

## 3. Research results

**Figure 2:**
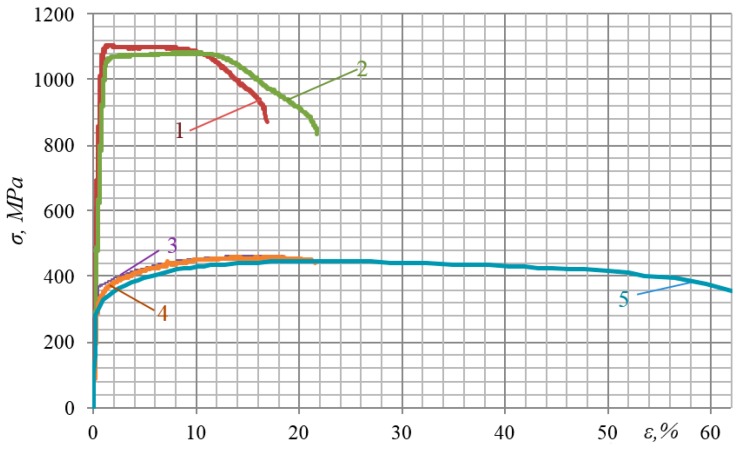
Stress-strain diagrams of materials: 1—Titanium alloy VT23; 2—titanium alloy VT23M; 3—aluminum alloy 2024-T351; 4—aluminum alloy D16ChATW; 5—stainless steel 12Kh17.

Figure 2 presents stress-strain diagrams for the materials investigated. As seen from these diagrams, the materials have essentially different mechanical properties. Therefore, it is clear that under practically the same additional force impulse loading applied to specimens (42.0 ± 2.0 kN), sudden increases in strain under the impulse introduction of energy *ε_imp_* will vary significantly. The results of testing three specimens from each alloy were as follows. The mean value of *ε_imp_* was 6.33% for aluminum alloy D16ChATW; 6.01% for alloy 2024-T351, 0.85% for alloy VT23, and 0.82% for alloy VT23M.

**Figure 3:**
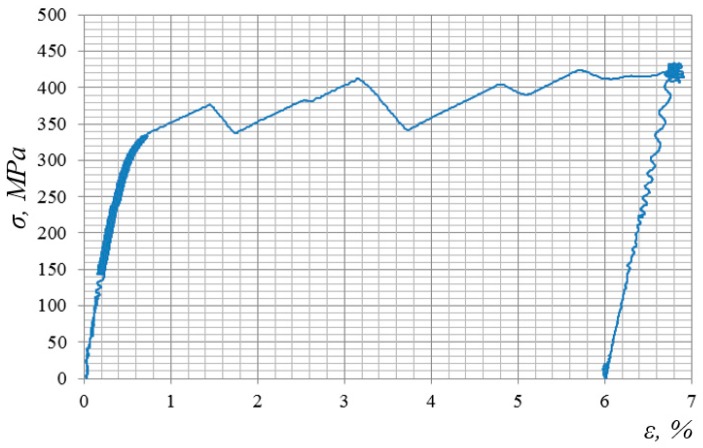
Stress-strain diagram of alloy 2024-T351 in the process of impulse introduction of force energy.

Figure 3, for instance, shows a stress-strain diagram for the alloy 2024-T351 obtained in the process of impulse introduction of energy. The very nature of the diagram shows the complex physical processes that occur in the alloy under impact-oscillatory loading.

As shown in previous studies, the physical nature of the dissipative structures depends both on the initial mechanical properties of the materials and on their chemical composition under DNP. It is interesting to trace changes in the topography of surface layers of materials after DNP. Impulse energy *ε_imp_* was introduced under the same force impulse loading applied to different materials. As a result, the range of sudden increases in strain differed significantly. Therefore, one can expect various effects from the changes in topography of the surface layers depending on the value of *ε_imp_*.

**Figure 4:**
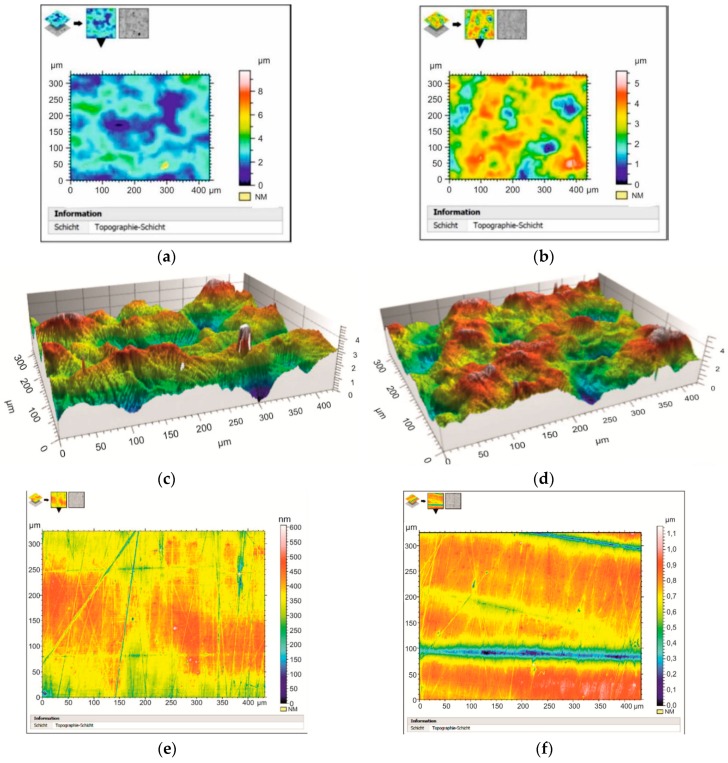

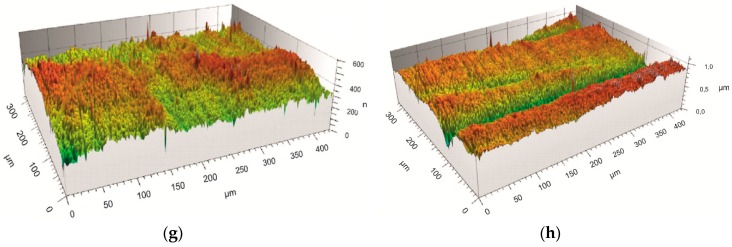
Topography of flat surfaces of specimens from alloys D16ChATW (**a**,**c**) and 2024-T351 (**e**,**g**) in the initial state (**a**,**c**,**e**,**g**) and after DNP (**b**,**d**,**f**,**h**): (**a**,**b**,**e**,**f**)–two-dimensional (2D)-measurements; (**c**,**d**,**g**,**h**)–three-dimensional (3D)-measurements.

**Figure 5:**
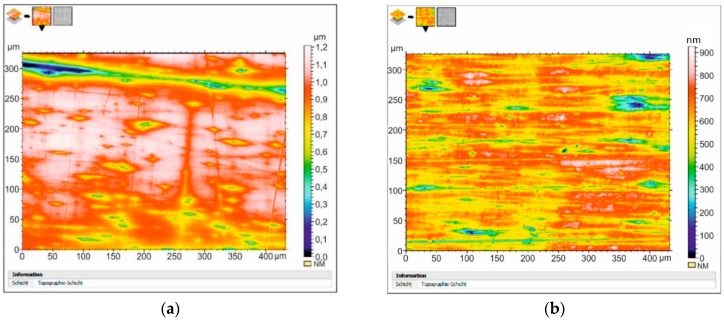

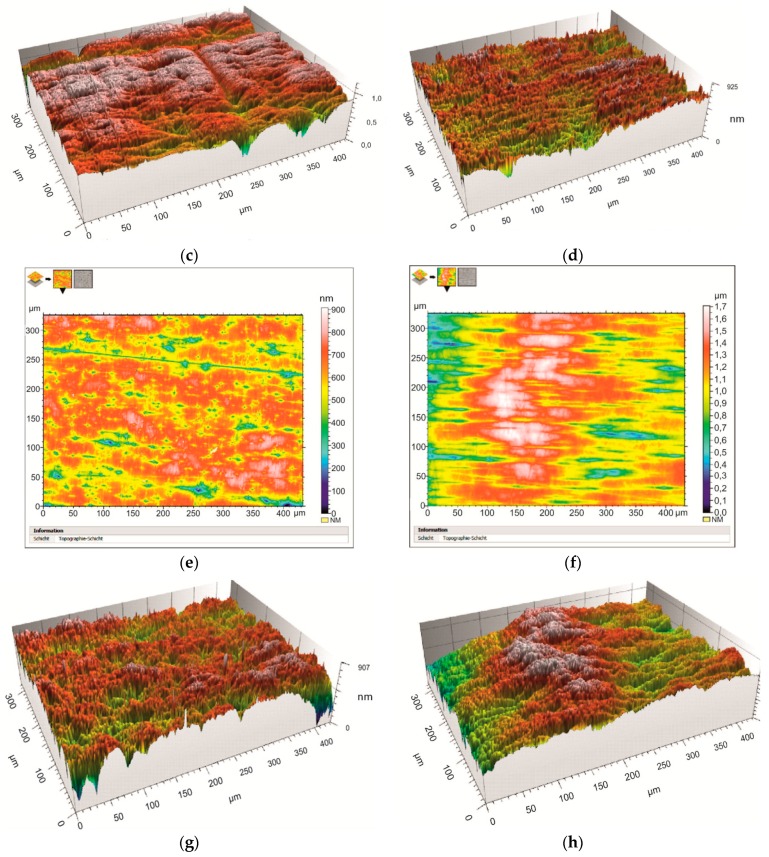
Topography of flat surfaces of specimens from alloys VT23 (**a**,**c**) and VT23M (**e**,**g**) in the initial state (**a**,**c**,**e**,**g**) and after DNP (**b**,**d**,**f**,**h**): (**a**,**b**,**e**,**f**)—2D-measurements; (**c**,**d**,**g**,**h**)—3D-measurements.

**Figure 6:**
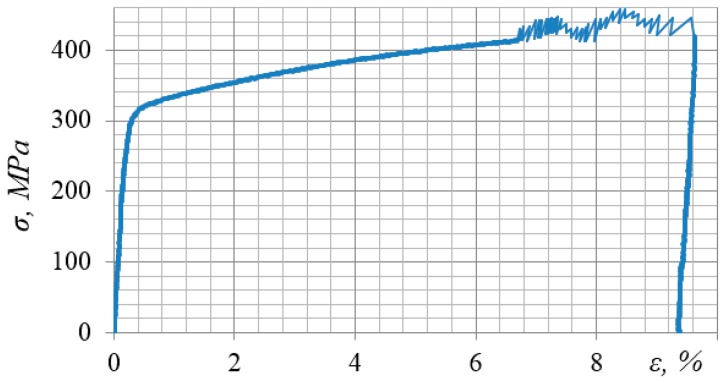
Stress-strain diagram of steel 12Kh17 in the process of preliminary static loading and in the process of impulse introduction of energy (F_imp_ = 120 kN).

**Figure 7:**
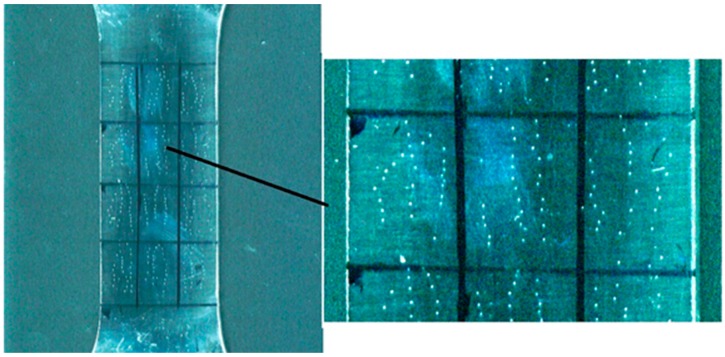
Grid applied to the specimen for hardness measurement and the location of inclusions.

**Figure 8:**
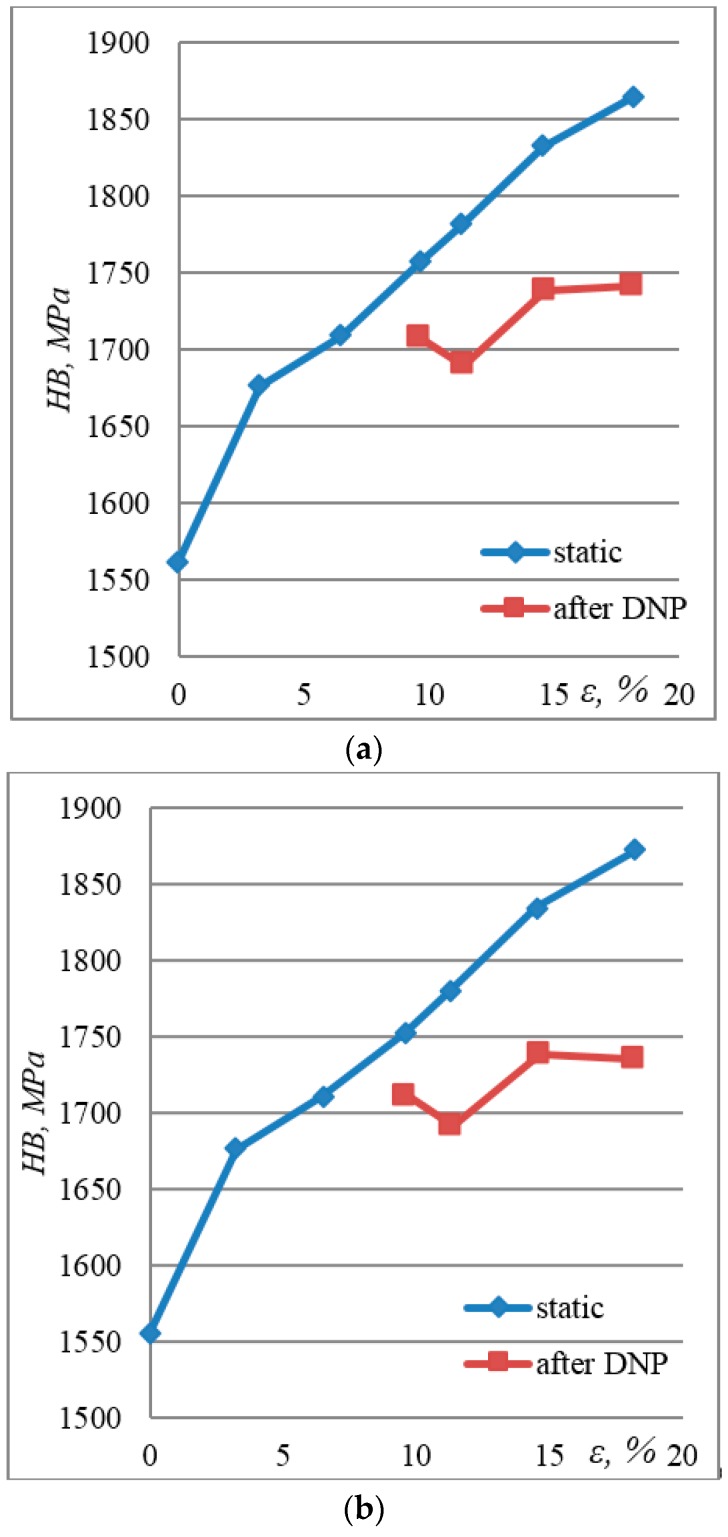

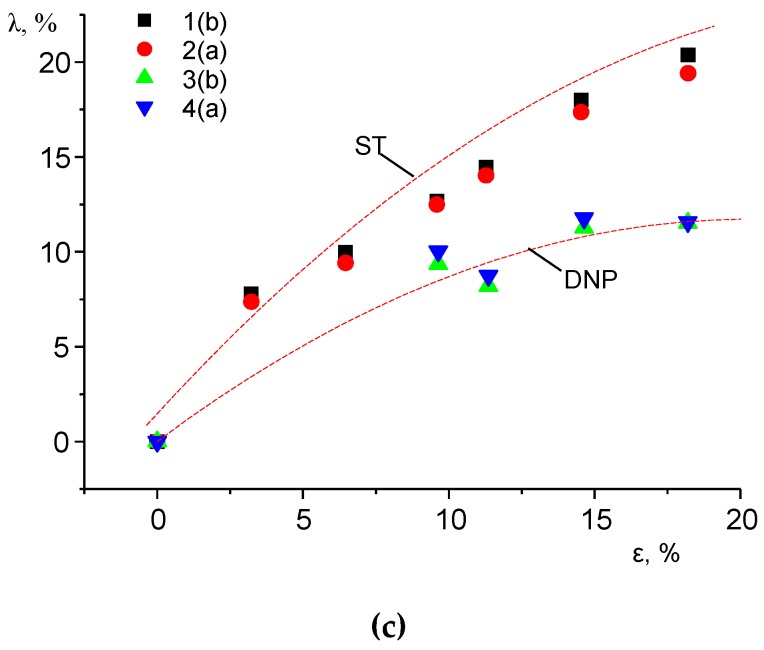
Distribution of hardness on surface layers of steel 12Kh17 depending on residual strain: (**a**) Average value throughout the gauge length of the specimen; (**b**) average value on the central rectangle (F_imp_ = 120 kN); (**c**) values of λ (%) after ST (1,2) and DNP (3,4).

Figure 4, Figure 5, Figure 6, Figure 7 and Figure 8 present the results of the quantitative evaluation of changes in the topography of flat surfaces of the specimens in the initial state and after DNP using the 3D Optical Surface Metrology System Leica DCM8. In all cases, the images are arranged in the direction of the axial loading. For clarity, the results obtained for each material are presented and analyzed in pairs. Here, Figure 4a,c,e,g and Figure 5a,c,e,g correspond to specimens in the initial state, and Figure 4b,d,f,h and Figure 5b,d,f,h correspond to specimens subjected to impact-oscillatory loading. Initially, general features of changes in the topography of surfaces for each pair of specimens from different materials were analyzed. Then, the roughness parameters R_z_ and R_a_ for the materials in the initial state and after the impulse introduction of energy were compared and summarized in Table 3. The surface roughness parameters were determined according to the roughness standard DIN EN ISO 4287: 2010. Data were calculated based on the “extraction” of a profile with the length of 300 µm parallel to the load axis. Along the metered topography, three lines were drawn conventionally, on which the profile was taken. The lines were spaced approximately evenly in the top, middle, and bottom of the topography. Figure 4 presents the data obtained for aluminum alloys D16ChATW and 2024-T351 (*ε_imp_* = 6.0–6.3%).

Let us note that the topography of flat surfaces of the two aluminum alloys analyzed is very different in the initial state. However, after DNP, the structures formed on the surfaces of alloys D16ChATW and 2024-T351 have both similarities and differences. Thus, the uniformity of the relief profile increases significantly after DNP in both alloys (see Figure 4d and Figure 5d). However, in addition to a regular system of microextrusions that appeared in the alloy 2024-T351, an orderly fibrous structure was also formed along the axis of loading (Figure 5d).

Figure 5 presents the research findings for specimens from titanium alloys. The consequences of impact-oscillatory loading for alloy VT23 (*ε_imp_* = 0.85%) manifest themselves, first of all, in the straightened relief and newly-formed fibrous structure (see Figure 5c,d). The signs of the regular ordered system of microextrusions that appeared in the relief are clearly evident. For alloy VT23M (*ε_imp_* = 0.82%), the effects of changes in the topography of flat specimen surfaces after DNP are more striking. In addition, a fibrous relief structure is created after DNP. The significant difference in the variation of the relief topography for alloys VT23 and VT23M can be explained by a significant difference in the percentage composition of α and β phases in the alloys. In the titanium alloy VT23, the β-phase is 43 wt.%, α-phase is 57 wt.%; in the titanium alloy VT23M, the β-phase is 22 wt.%, and α-phase is 78 wt.% [34].

When analyzing the experimental data obtained for all materials, it can be clearly stated that with any intensity of the impulse introduction of force energy into materials, the effects from the varying relief topography due to the appearance of dissipative structures under DNP are pronounced. The roughness parameters R_z_ and R_a_ were chosen as the main parameters that characterize changes in the relief (Table 3). In future, these features can be used to determine the optimal mode of impulse introduction of energy into materials in order to maximize the fatigue life of alloys.

The analysis of Table 3 shows that the relief topography changes significantly after DNP in all the materials investigated. Changes in the surface morphology are a good indicator of the internal state of materials, which allowed obtaining information on the presence/absence of damage and will be subsequently used to describe the condition of the structure [35,36,37,38,39,40]. This is manifested in the alignment of the relief profile. For instance, for the D16ChATW alloy, the unevenness of the profile relief R_z_ in the studied region was 39.8% in the initial state and 23.3% after DNP. For alloy VT23, they were 50% and 27.3%, respectively. For alloy VT23M, they were 25.1% and 11.8%, respectively. On the other hand, for alloy 2024-T351, the unevenness of the relief profile increased after DNP from 18.7% to 37.5%. This is due to the appearance of a fibrous structure in the longitudinal direction (see Figure 5d).

A similar assessment of the effect caused by DNP on the roughness parameter R_a_ in the studied region showed that this value increased only slightly for alloy VT23M from 11.1% in the initial state to 12.5% after DNP. For all other materials, these values were decreased after DNP. For alloy D16ChATW, a decrease was from 35.5% to 26.0%. For alloy 2024-T351, these values were decreased from 21.2% to 20.9%. For alloy VT23, a decrease was from 61.1% to 31.1%. A comparative quantitative assessment of the surface roughness of the investigated materials in the initial state and after DNP also revealed significant differences. To evaluate changes in the hardness of surface layers after DNP, specimens (Figure 1b) from steel 12Kh17 were used. Research was conducted in three stages. In the first stage, four specimens were tensioned to a certain value of static strain (6.68%; 8.03%; 11.68%; 14.42%); then they were subjected to DNP under additional force loading 120 ± 4 kN and unloaded immediately.

Figure 6 presents a stress-strain diagram for steel 12Kh17 in the process of preliminary static loading and in the process of impulse introduction of energy at the static strain level of 6.68%. When analyzing Figure 6, two important points can be noted. Firstly, with the impulse introduction of force energy into the material, a high-frequency cyclic loading is realized. Secondly, in some strain ranges, a drop in strength is observed during DNP. This can be directly related to the formation of less dense dissipative structures.

In the second stage, four similar specimens were tensioned statically. The level of residual strains was recorded at a complex loading mode “static tension—DNP” (9.64%; 11.36%; 14.64%; 18.20%). To construct a diagram showing the dependence of hardness on the level of residual strains under static loading, specimens were tensioned statically to two levels of strain: 3.23% and 6.46%. In the third stage, all tested specimens, including a completely unstrained specimen, were polished on one side, and a grid with 12 identical rectangles was applied to the polished part (Figure 7). Then, using the Computest SC portable hardness meter, minimum 27 hardness tests on the HB scale were performed in each of the rectangles (see Figure 7).

The ambivalent nature of a decrease in ductility under the constant yield strength and a slight decrease in strength are associated with several effects applied under DNP:

1. It should be emphasized that steel hardness, compared with the initial state, increases both after static tension and DNP, but with different intensities, Figure 8a,b. This fully agrees with the physical concepts of strain hardening of materials [41,42,43,44,45,46]. Parameter λ = (HVi − HV_0_)/HV_0_ × 100% was used to calculate variations in surface hardness after static tension (ST) and DNP + ST. The following values were obtained, Figure 8c. The ε—λ curves have a similar shape, but different curvatures depending on the deformation conditions. This is due to the difference in the mechanisms that control the plastic flow of the material [47,48,49]. In our opinion, when grain `boundaries are improved in the process of DNP, this leads to a decrease in the intensity of surface hardening [50,51]. This fact indicates the presence of relaxation processes on the surface, as well as annihilation and redistribution of dislocations in the surface layers of the material [47,51]. This assumption is confirmed by the results of previous studies, as well as literature data [49]. Thus, at ε = 10–18.2%, the value of λ was from 13.9% to 20.1% for ST, and from 8.7% to 11.7% for DNP.
2. The static deformation of steel is of the “classical” type, because surface hardening occurs due to the accumulation of dislocations in the low-angle grain boundaries [52]. This aspect has not been considered in detail, since the analysis of dislocation structures of such steels is described in ref. [53].
3. Steels are usually characterized by a tendency to localize strains. As a result, larger volumes of the material are involved in plastic deformation, and a lengthy stage of strain hardening is preserved. The latter allows maintaining a high ultimate strength, albeit less than the initial. Subsequently, the localized accumulation of structural defects leads to a decrease in ductility, which was observed in our case.
4. In contrast to the volume of the material, the preceding plastic flow in the surface layer causes the formation of a folded surface structure (microextrusions). In zones of a pronounced local curvature, dislocations appear in the folds, which penetrate deep into the material. As a result, the surface becomes inhomogeneous, because surface irregularities are generally “softer” than those of the base material [54]. Under such conditions, the surface layer is an effective generator of dislocations [47,55]. As a result, small folds disappear on the surface and large ones become smoother, which accelerates the macrolocalization of strain and leads to the fracture of the specimen. It is these factors that cause a decreased ductility of steel 12Kh17 under a constant yield strength and a slight decrease in strength during DNP.

The results obtained are intended, primarily, for aircraft and machine building, as well as for the strength calculation of metal parts that are sensitive to the accumulation of plastic strains, for instance, under various types of impact loading.

## 4. Conclusions

A quantitative evaluation of the relief topography of surface layers of materials of different classes after dynamic deformations in the process of DNP has been conducted, and hardness of surface layers of the material after DNP was evaluated.

After the realization of DNP, practically all materials showed the signs of a regular ordered system of microextrusion formed along the axis of loading throughout the relief. In addition, DNP affects the alignment of roughness parameters R_z_ and R_a_ of the relief profile. For the D16ChATW alloy, the unevenness of the profile roughness R_z_ in the studied region was 39.8% in the initial state and 23.3% after DNP. For alloy VT23, they are 50% and 27.3%, respectively. For alloy VT23M, they are 25.1% and 11.8%, respectively. On the other hand, for alloy 2024-T351, the unevenness of the profile roughness increased after DNP from 18.7% to 37.5%. This is due to the appearance of a fibrous structure in the longitudinal direction.

It is interesting to note that for all the materials under investigation, the absolute values of roughness parameters (R_z_) decreased after DNP, even with a significant increase in the dynamic strain (*ε_imp_* = 6.3%). This contradicts the well-known reports about an increase in roughness parameter R_z_ with an increase in the strain under static tension. Similar effects are also manifested in the alignment of the roughness parameter R_a_ of the relief profile. Only for the VT23M alloy, this value increased slightly after DNP. For all other materials, these values decreased significantly after DNP.

Hardness measurements indicate that for the surface hardening of steel 12Kh17, DNP is less effective than the static tension. Thus, compared to the initial hardness (HB) of 1555, the average hardness of the alloy was 1872 after ST and 1735 after DNP at the same level of strain (ε = 18.2%).

In contrast to the volume of the material, the preceding plastic flow in the surface layer causes the formation of a folded surface structure (microextrusions), leading to an increased strain localization under DNP. The static tension is characterized by “classical” deformation, with surface hardening due to the accumulation of dislocations within the low-angle grain boundaries.

## Figures and Tables

**Table 1:** Mechanical properties of materials studied.

Materials	σ_ys_, MPa	σ_us_, MPa	δ, %
Aluminum alloy 2024-T351	342	462	20.5
Aluminum alloy D16ChATW	322	452	21.5
Titanium alloy VT23	980	1100	16.0
Titanium alloy VT23M	1000	1080	21.0
Stainless steel 12Kh17	280	445	62.0

**Table 2:** Chemical composition of materials studied.

**Aluminum alloy, %**	**Si**	**Fe**	**Cu**	**Mn**	**Mg**	**Cr**	**Zn**	**Ti**
2024-T351	0.05	0.13	4.7	0.70	1.5	0.01	0.02	0.04
D16ChATW	0.11	0.18	4.4	0.63	1.4	0.01	0.01	0.07
**Titanium alloy, %**	**Fe**		**Cr**	**Mo**		**V**	**Ti**	**Al**
VT23	0.6		1.2	2.0		4.3	86.9	5.0
VT23M	0.7		1.1	2.2		4.5	86.7	4.8
**Stainless steel, %**	**C**	**Si**	**Mn**	**S**		**P**	**Cr**	**Fe**
12Kh17	≤0.12	≤0.8	≤0.8	≤0.025		≤0.035	16.0–18.0	~81.0

**Table 3:** Roughness parameters R_z_ and R_a_ of specimen surfaces in the initial state and after impulse introduction of energy.

Material		Measurement Area of Profile with the Length of 300 µm	Roughness Parameter R_z_	Roughness Parameter R_a_
D16CzATW alloy	Initial state	Upper part of topography	554	126
		Medium part of topography	886	145
		Lower part of topography	533	93.5
	After DNP	Upper part of topography	757	178
		Medium part of topography	630	151
		Lower part of topography	822	204
Aluminum alloy 2024-T351	Initial state	Upper part of topography	165	18.9
		Medium part of topography	182	14.9
		Lower part of topography	203	18.7
	after DNP	Upper part of topography	130	17.2
		Medium part of topography	208	21.7
		Lower part of topography	164	20.6
Titanium alloy VT23	Initial state	Upper part of topography	113	15.1
		Medium part of topography	202	34.2
		Lower part of topography	101	13.3
	After DNP	Upper part of topography	154	18.2
		Medium part of topography	112	15.7
		Lower part of topography	154	22.8
Titanium alloy VT23M	Initial state	Upper part of topography	246	32
		Medium part of topography	194	33
		Lower part of topography	259	36
	After DNP	Upper part of topography	150	28.6
		Medium part of topography	167	31.9
		Lower part of topography	170	32.7
